# Incidence and Management of Life-Threatening Adverse Events During Cardiac Catheterization for Congenital Heart Disease

**DOI:** 10.1007/s00246-013-0752-y

**Published:** 2013-07-31

**Authors:** C. Huie Lin, Sanjeet Hegde, Audrey C. Marshall, Diego Porras, Kimberlee Gauvreau, David T. Balzer, Robert H. Beekman, Alejandro Torres, Julie A. Vincent, John W. Moore, Ralf Holzer, Laurie Armsby, Lisa Bergersen

**Affiliations:** 1 Methodist DeBakey Heart and Vascular Center, Houston, TX USA; 2 Rady Children’s Hospital, San Diego, CA USA; 3 Boston Children’s Hospital, Boston, MA USA; 4 St. Louis Children’s Hospital, St. Louis, MO USA; 5 Cincinnati Children’s Hospital Medical Center, Cincinnati, OH USA; 6 Morgan Stanley Children’s Hospital of New York—Presbyterian, New York, NY USA; 7 Nationwide Children’s Hospital, Columbus, OH USA; 8 Oregon Health and Science University, Portland, OR USA; 9 6550 Fannin Street, Suite 1901, Smith Tower, Houston, TX 77030 USA

**Keywords:** Cardiac catheterization and intervention, Mortality, ECMO, Cardiac surgery, Congenital heart disease

## Abstract

Continued advancements in congenital cardiac catheterization and interventions have resulted in increased patient and procedural complexity. Anticipation of life-threatening events and required rescue measures is a critical component to preprocedural preparation. We sought to determine the incidence and nature of life-threatening adverse events in congenital and pediatric cardiac catheterization, risk factors, and resources necessary to anticipate and manage events. Data from 8905 cases performed at the 8 participating institutions of the Congenital Cardiac Catheterization Project on Outcomes were captured between 2007 and 2010 [median 1,095/site (range 133–3,802)]. The incidence of all life-threatening events was 2.1 % [95 % confidence interval (CI) 1.8–2.4 %], whereas mortality was 0.28 % (95 % CI 0.18–0.41 %). Fifty-seven life-threatening events required cardiopulmonary resuscitation, whereas 9 % required extracorporeal membrane oxygenation. Use of a risk adjustment model showed that age <1 year [odd ratio (OR) 1.9, 95 % CI 1.4–2.7, *p* < 0.001], hemodynamic vulnerability (OR 1.6, 95 % CI 1.1–2.3, *p* < 0.01), and procedure risk (category 3: OR 2.3, 95 % CI 1.3–4.1; category 4: OR 4.2, 95 % CI 2.4–7.4) were predictors of life-threatening events. Using this model, standardized life-threatening event ratios were calculated, thus showing that one institution had a life-threatening event rate greater than expected. Congenital cardiac catheterization and intervention can be performed safely with a low rate of life-threatening events and mortality; preprocedural evaluation of risk may optimize preparation of emergency rescue and bailout procedures. Risk predictors (age < 1, hemodynamic vulnerability, and procedure risk category) can enhance preprocedural patient risk stratification and planning.

**Electronic supplementary material:**

The online version of this article (doi:10.1007/s00246-013-0752-y) contains supplementary material, which is available to authorized users.

## Introduction

Significant advances in transcatheter technologies [1, 19] have increased the complexity and heterogeneity of congenital cardiac catheterization procedures as well as the complexity and pre-existing morbidity of patients undergoing catheterization. Previous studies of pediatric cardiac catheterization reported rates of major complications of 0.9–6 % and mortality of 0.14–0.7 %; however, these reports originated from single-center retrospective studies with heterogeneous case mixes and data predating technological advances of the current era [9, 17, 18]. Work from this group has previously shown the overall rate of life-threatening adverse events during congenital and pediatric cardiac catheterization and predictors of these events [4]. As quality improvement efforts [10], mandatory registries [14], and appropriate use criteria [15] evolve for cardiac catheterization, proactive interventionalist-driven definitions of standards of care have become a priority. Similarly, necessary resources for hemodynamic support, transcatheter, and surgical rescue based on preprocedural risk stratification must be delineated.

The purpose of this study was to use prospectively collected data from the multicenter Congenital Cardiac Catheterization Project on Outcomes (C3PO) study to report the incidence and specific nature of life-threatening adverse events during congenital catheterization, define risk factors to anticipate events, and describe the emergency rescue procedures required to manage these events. In addition, the previously developed Catheterization for Congenital Heart Disease Adjustment for Risk Method (CHARM) was applied to report standardized life-threatening event ratios by institution.

## Methods

Data were prospectively collected for the C3PO study beginning on February 1, 2007, at six centers, adding two additional centers in May 2008 and July 2009 through January 31, 2010. Participating centers recorded patient and procedural characteristics and the occurrence of adverse events using a Web-based data entry tool as previously described [4]. Boston Children’s Hospital was the sponsor and data coordinating center for the project. All diagnostic or interventional catheterizations were included in the analysis, whereas hybrid procedures and biopsies were excluded due to the low incidence of life-threatening events as previously reported [4, 12]. Data collection, validation, and auditing has previously been reported [4] and has confirmed accurate and complete capture of serious adverse events in the database. Established nomenclature was used to classify adverse event severity ranging from severity levels 1 to 5 (Supplemental Table 1) as well as adverse-event preventability (Supplemental Table 2) [2, 5–7, 10, 12]. All events were reviewed by two designated physicians for proper classification of event severity and preventability, and any discrepancies with the sites preliminary designation were resolved prospectively during the study. Life-threatening events were defined as (1) adverse events related to the catheterization procedure, (2) identified during or after the procedure resulting in a change in patient condition, (3) life-threatening if not treated, (4) requiring major intervention, such as invasive monitoring or major transcatheter bailout procedure (severity level 4), and (5) resulting in death and emergency surgery or failure to wean from extracorporeal membrane oxygenation (severity level 5, Supplemental Table 1).

## Statistical Analysis

The incidence of life-threatening events was calculated for the study population; frequency and percent were calculated for adverse event details, type, attributability, severity, preventability, and management. Patient and procedural characteristics were compared for subjects with and without a life-threatening adverse event using Chi square test for categorical variables and Wilcoxon rank-sum test for continuous variables.

A multivariable model for the outcome serious adverse events (levels 3 through 5) has previously been described [8]; three independent predictors are included in CHARM, namely, age, hemodynamic vulnerability (Supplemental Table 3) [8], and procedure type risk category (Supplemental Table 4) [5]. The relationship between these factors and occurrence of life-threatening adverse events (levels 4 and 5) was evaluated using logistic regression analysis; odd ratios (ORs) and 95 % Confidence intervals (CIs) are reported.

The risk-adjusted expected frequencies of life-threatening events were calculated for each institution using CHARM [3, 8]. Standardized life-threatening event ratios were calculated by dividing each institution’s observed life-threatening event rate by this expected event rate. A standardized life-threatening event ratio of 1.0 indicated that the observed event rate is equal to the expected rate given the institution’s case mix complexity; 95 % CIs were calculated for each event ratio.

## Results

### Incidence and Characteristics of Life-Threatening Events

**Fig. 1:**
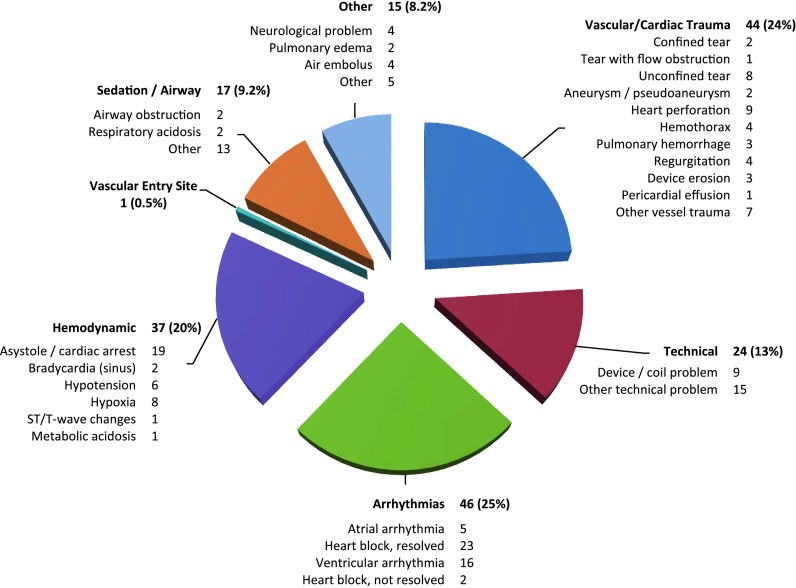
Categories of life-threatening adverse events (severity levels 4 and 5) during cardiac catheterization. Percentage and individual event rates are presented

Between February 2007 to January 2010 (36 months), 8905 cases were captured at 8 sites in the C3PO registry [median 1,095 cases/site (range 133–3,802)]. During this period, 188 life-threatening (levels 4 or 5) events occurred in 184 patients (2.1 %, 95 % CI 1.8–2.4 %). Patient and procedural characteristics are listed in Tables 1 and 2. In terms of types of events, 25 % of the life-threatening events were related to cardiac arrhythmia, 24 % to cardiac or vascular trauma, 20 % to hemodynamic instability, 13 % to device, coil, stent or other technical issues, 9.5 % to sedation/anesthesia or airway, 8 % to neurological complications, air embolus, and pulmonary edema, or other issues, and 0.5 % to vascular entry site (Fig. 1).

**Table 1:** Patient and procedural characteristics

Patient and Case characteristics	Life threatening adverse event (*n* = 184) *N* (%) or median [IQR]	No life threatening event (*n* = 8,721) *N* (%) or median [IQR]	*P* value
Age			<0.001
<1 month	45 (25 %)	743 (9 %)	
1 to 11 months	53 (29 %)	1920 (22 %)	
1 to 10 years	51 (28 %)	3417 (39 %)	
≥11 years	35 (19 %)	2632 (30 %)	
Not recorded	0 (0 %)	9 (<1 %)	
Weight (kg)	7 [3.7, 18.7]	14.2 [6.7, 42.3]	<0.001
Case Type			<0.001
Interventional	147 (80 %)	5692 (65 %)	
Diagnostic	37 (20 %)	3029 (35 %)	
Diagnosis			0.02
No structural heart disease (i.e. myopathy)	9 (5 %)	455 (5 %)	
Pulmonary hypertension	3 (2 %)	333 (4 %)	
Isolated defects	32 (17 %)	2325 (27 %)	
Complex defect with two ventricles	81 (44 %)	3435 (39 %)	
Complex defect with one ventricle	59 (32 %)	2170 (25 %)	
Not recorded	0 (0 %)	3 (<1 %)	
Genetic syndrome	23 (13 %)	1216 (14 %)	0.71
Non-cardiac problem	57 (31 %)	2465 (28 %)	0.31
Surgery in prior 30 days	20 (11 %)	531 (6 %)	0.01
Hemodynamic indicators of vulnerability			
Mixed venous saturation <60 % two ventricle or <50 % single ventricle	52 (28 %)	1369 (16 %)	<0.001
Systemic ventricle end diastolic pressure ≥18 mmHg	21 (11 %)	480 (6 %)	0.002
Systemic arterial saturation <95 % non-single ventricle or <78 % single ventricle	88 (48 %)	2747 (32 %)	<0.001
Main pulmonary artery pressure systolic ≥45 mmHg non-single ventricle, mean ≥17mmHg single ventricle	46 (25 %)	1621 (19 %)	0.04
Number of hemodynamic indicators			<0.001
0	68 (37 %)	4644 (53 %)	
1	49 (27 %)	2336 (27 %)	
≥2	67 (36 %)	1741 (20 %)	
Admission source			<0.001
Elective	90 (49 %)	6719 (77 %)	
Non-elective	71 (39 %)	1824 (21 %)	
Emergent	23 (13 %)	177 (2 %)	
Not recorded	0 (0 %)	1 (<1 %)	
Transferred on ECMO support	11 (6 %)	128 (1 %)	<0.001
Spontaneous respirations	17 (9 %)	2217 (25 %)	<0.001
Procedure type risk group			<0.001
Group 1	18 (10 %)	2078 (24 %)	
Group 2	30 (16 %)	3221 (37 %)	
Group 3	60 (33 %)	2051 (24 %)	
Group 4	61 (33 %)	1127 (13 %)	
Unable to be assigned	15 (8 %)	244 (3 %)	
Inotropic support during the case	87 (47 %)	1121 (13 %)	<0.001
Case duration			<0.001
<1 hour	23 (13 %)	1916 (22 %)	
≥1, <3 hours	125 (68 %)	5995 (69 %)	
≥3, <4 hours	20 (11 %)	590 (7 %)	
≥4 hours	16 (9 %)	199 (2 %)	
Not recorded	0 (0 %)	21 (<1 %)	
Contrast dose (cc/kg)	4.5 [2.4, 6.0]	3.3 [1.7, 5.2]	<0.001

^a^Data are shown as *N* (%) or median (IQR)

Fifty-seven percent of the cases with life-threatening events required cardiopulmonary resuscitation (CPR), and 13 % required electrical cardioversion. Nine percent of patients (*n* = 16) who experienced life-threatening events required ECMO support (details in Supplemental Table 5), and 44 % of these patients (*n* = 7) ultimately died despite support. In 10 % of life-threatening events, emergent percutaneous interventions were required, including pericardiocentesis (*n* = 12), thoracentesis (*n* = 2), and vessel coil occlusion (*n* = 2). Fifteen percent of life-threatening adverse events required emergent surgery (*n* = 28) for management. Reasons for emergent surgery included device extraction for malposition (*n* = 1), device embolization (*n* = 5) or erosion (*n* = 3), stent embolization (*n* = 5), vessel trauma (*n* = 5), unconfined vascular tear (*n* = 3), and injury to mitral valve structure (*n* = 2). Attempted surgical rescue was unsuccessful in 5 patients.

Of the 188 life-threatening events, 25 were catastrophic (level 5) with an overall procedural mortality rate of 0.28 % (95 % CI 0.18–0.41 %). Event details are listed in Supplemental Table 6. Of note, 5 deaths resulted from cardiac perforation during atrial septostomy: 1 adult patient with end-stage pulmonary hypertension for atrial septal defect creation with the remaining 4 in neonates with single or complex two-ventricle physiology and intact atrial septa. Four other neonates died as a result of unsuccessful atrial septostomy or pre-existing profound hemodynamic derangement before transfer despite successful intervention. Indeed, 10 of the 25 deaths occurred in patients who were ≤10 days old.

One death resulted from a pulmonary hypertensive crisis after successful atrial septal defect creation; one patient arrested after dissection complicating selective coronary angiography during transcatheter pulmonic valve implantation; one lung transplant patient with pulmonary vein stenosis, who was moribund, died from hypotension degenerating to cardiac arrest after pulmonary venoplasty; and one patient arrested due to left main coronary stent thrombosis. One patient died from complications of small-bowel infarction after embolization of a coil to the superior mesenteric artery occurred while attempting to coil-occlude a patent ductus arteriousus stent. Finally, one patient arrested after aspiration of formula after the procedure and on postmortem examination was found to have a large retroperitoneal hemorrhage due to attempted femoral access. A complete list of cases requiring ECMO are given in Supplemental Table 5. Cases resulting in death are listed in Supplemental Table 6.

Preventability of each life-threatening event was classified by the procedural physician and reviewed during the described data audit by two independent physicians. Of the life-threatening events, 10.1 % (*n* = 19) were classified as “preventable,” suggesting a definite breech of standard technique or necessary precautions being not taken and thus that the event was preventable by modification of technique or care. For example, in one case, air was inadvertently injected during left ventriculography with air seen in the anterior sinus of Valsalva, resulting in hypotension and bradycardia with a heart rate approximately 30 beats/min requiring CPR followed by direct current cardioversion. After these resuscitation maneuvers, the patient recovered, and the remainder of the procedure was completed uneventfully.

In contrast, 47.3 % of cases were classified as “possibly preventable” (*n* = 89), whereas 42.6 % of life-threatening events (*n* = 80) were classified as “not preventable,” thus suggesting that no clearly known alteration in method or care existed to prevent the event. One example of a not-preventable adverse event involved a critically ill single-ventricle patient status post-Glenn who arrived in the catheterization laboratory pulseless and apneic with severe respiratory acidosis and severe ventricular dysfunction. Efforts to resuscitate the patient were unsuccessful; however, a single venogram was performed successfully and showed no structural abnormality in the Glenn circuit. The patient subsequently died.

### Risk Factors for Life-Threatening Adverse Events

**Fig. 2:**
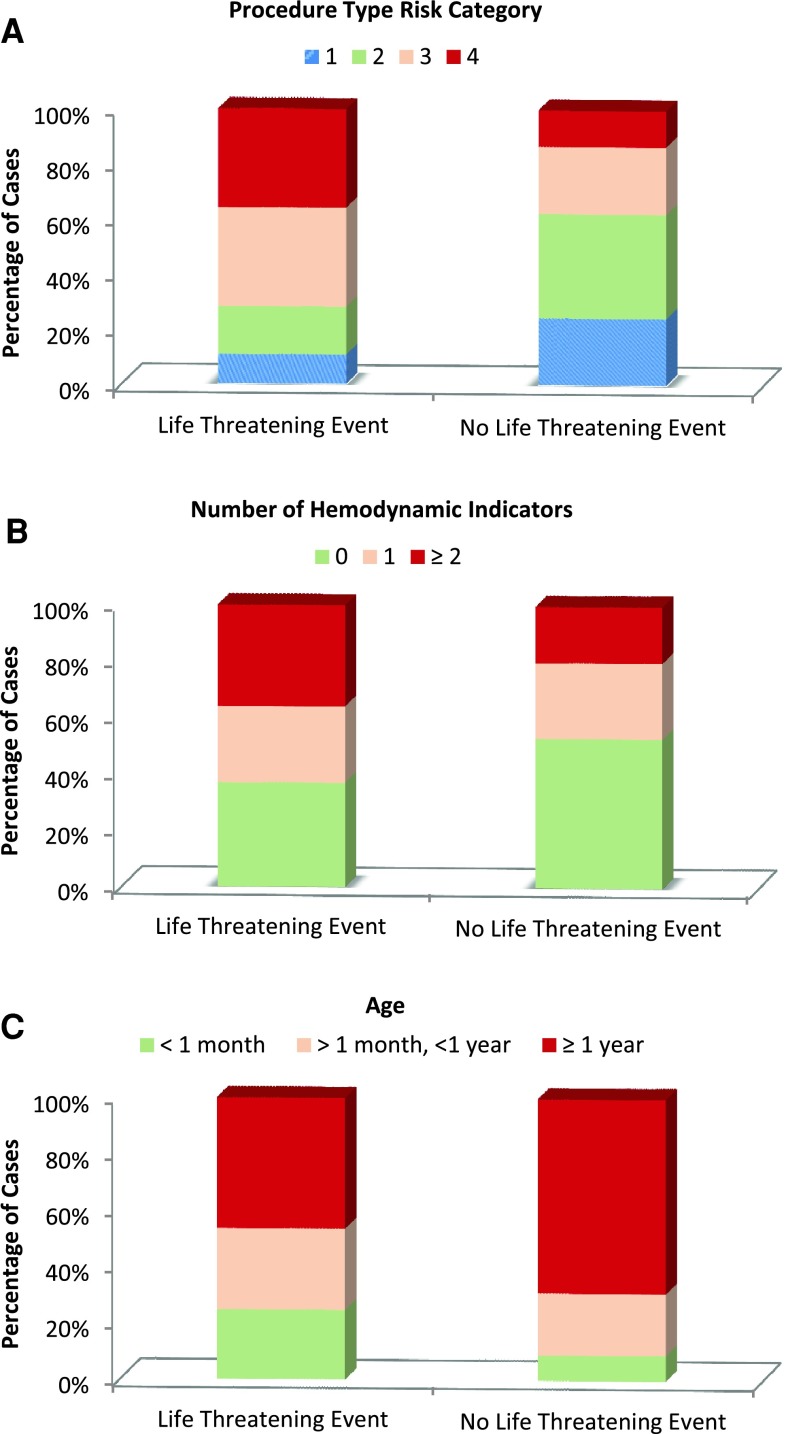
Distribution of risk factors for subjects with and without life-threatening events. **a** Procedure-type risk category. **b** Number of hemodynamic indicators. **c** Age

Univariate analysis of possible predictors of life-threatening adverse events was performed (Table 2) and showed that age < 1 year, recent cardiac surgery, ≥2 hemodynamic indicators of vulnerability, transfer on ECMO support, high-risk procedure, and longer case duration were potential predictors. The multivariable CHARM [8] model was applied to the outcome “life-threatening adverse events” and the magnitudes of the effect of age < 1 year, hemodynamic indicators of increased vulnerability, and procedure type risk category estimated (Table 3). Specifically, children < 1 year of age undergoing catheterization had nearly twice the odds of life-threatening events (OR 1.9, 95 % CI 1.4–2.7, *p* < 0.001) relative to patients >1 year. Second, patients ≥ 2 or more indicators of hemodynamic vulnerability [8] (Supplemental Table 3) had greater odds of life-threatening events during cardiac catheterization with an OR of 1.6 (95 % CI 1.1–2.3, *p* < 0.01). Third, high-risk procedures [5] (Supplemental Table 4) were associated with double and quadruple the odds of life-threatening events; the OR was 2.3 (95 % CI 1.3–4.1, *p* = 0.003) for category 3 procedures and 4.2 (95 % CI 2.4–7.4, *p* < 0.001) for category 4 procedures relative to category 1. Percent of cases with and without life-threatening events with these three predictors is plotted in Fig. 2.

**Table 2:** Predictors of life-threatening events: univariate analysis

Predictors (%)	No. of patients	Life-threatening events	
		No. of events (%)	OR (95 % CI)
Age (years)			
<1	2761	98 (3.6)	2.6 (1.9, 3.5)
≥1	6135	86 (1.4)	1.0
Diagnosis			
No structural heart disease	464	9 (1.9)	1.0
Pulmonary hypertension	336	3 (0.9)	0.5 (0.1, 1.7)
Isolated defects	2357	32 (1.4)	0.7 (0.3, 1.5)
Complex defect with 2 ventricles	3516	81 (2.3)	1.2 (0.6, 2.4)
Complex defect with 1 ventricle	2229	59 (2.7)	1.4 (0.7, 2.8)
Genetic syndrome	1239	23 (1.9)	0.9 (0.6, 1.4)
Noncardiac problem	2522	57 (2.3)	1.1 (0.8, 1.6)
Hemodynamic indicators of vulnerability			
0	4712	68 (1.4)	1.0
1	2385	49 (2.1)	1.4 (0.9, 2.1)
≥2	1808	67 (3.7)	2.6 (1.9,3.7)
Previous surgery < 30 days	551	20 (3.6)	1.9 (1.2, 3.0)
Transferred on ECMO support	139	11 (7.9)	4.3 (2.3, 8.0)
Spontaneous respirations	2234	17 (0.8)	0.3 (0.2, 0.5)
Procedure-type risk categories			
1	2096	18 (0.9)	1.0
2	3251	30 (0.9)	1.1 (0.6, 1.9)
3	2111	60 (2.8)	3.4 (2.0, 5.7)
4	1188	61 (5.1)	6.2 (3.7, 10.6)
Unable to be assigned	259	15 (5.8)	7.1 (3.5, 14.3)
Case duration (h)			
<1	1939	23 (1.2)	1.0
1–3	6120	125 (2.0)	1.7 (1.1, 2.7)
3–4	610	20 (3.3)	2.8 (1.5, 5.2)
>4	215	16 (7.4)	6.7 (3.5, 12.9)

**Table 3:** Catheterization for Congenital Heart Disease Adjustment for Risk Model (CHARM) Predictors and life-threatening adverse events

Predictors (%)	OR (95 % CI)	*P*
Age (years)		
<1	1.9 (1.4, 2.7)	<0.001
≥1	1.0	–
Hemodynamic indicators of vulnerability		
0	1.0	–
1	1.0 (0.7, 1.5)	0.87
≥2	1.6 (1.1, 2.3)	0.01
Procedure-type risk categories		
1	1.0	–
2	0.8 (0.4, 1.5)	0.46
3	2.3 (1.3, 4.1)	0.003
4	4.2 (2.4, 7.4)	<0.001
Unable to be assigned	5.7 (2.8, 11.5)	<0.001

### Life-Threatening Events and Risk Standardization

**Fig. 3:**
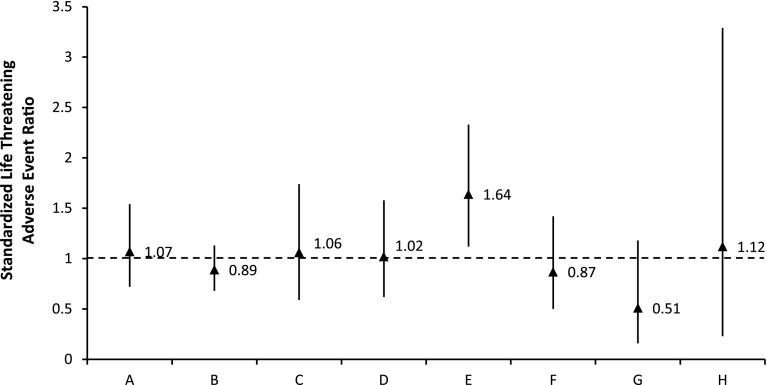
Standardized life-threatening adverse event ratios by institution. Standardized life-threatening adverse event ratios are plotted by institution (*triangles* error bars 95 % CI). Dashed line indicates observed life-threatening event rate = expected rate based on CHARM

Eight institutions contributed data with a median of 1,095 cases/site (range 133–3,802); the observed rate of life-threatening events per site ranged from 0.91 % to 3.27 %. The expected rates of life-threatening events (calculated based on CHARM) for each institution ranged from 1.73 to 2.35 % (Table 4). Standardized life-threatening event ratios (observed event rate/expected event rate) ranged from 0.51 to 1.64 (Fig. 3). One center (institution E) had a significantly greater standardized life-threatening event ratio, 1.64 (95 %CI 1.12–2.33). Of the life-threatening events, 6.5 % were reported as being preventable at this institution; other institutions reported between 0 and 17.7 % preventable life-threatening events, whereas the overall rate of preventable life-threatening events for the study was 10.1 %.

**Table 4:** Standardized life-threatening adverse event ratios for eight institutions

Hospital	No. (%) of life-threatening adverse events	No. of expected rate of life-threatening adverse events	Standardized life-threatening adverse event ratio (95 % CI)
A	29 (2.16)	2.01	1.07 (0.72, 1.54)
B	65 (2.08)	2.35	0.89 (0.68, 1.13)
C	15 (1.83)	1.73	1.06 (0.59, 1.74)
D	20 (2.13)	2.09	1.02 (0.62, 1.58)
E	31 (3.27)	1.99	1.64 (1.12, 2.33)
F	16 (1.53)	1.75	0.87 (0.50, 1.42)
G	5 (0.91)	1.79	0.51 (0.16, 1.18)
H	3 (2.17)	1.93	1.12 (0.23, 3.29)

## Discussion

This is the first work to describe a large multicenter experience with life-threatening events in congenital cardiac catheterization in the contemporary era. We report a low incidence of life-threatening events (2.1 %) and mortality (0.28 %) despite the complexity of modern patients and procedures. The majority of life-threatening events were treated successfully with CPR, ECMO, surgery, or percutaneous bail-out. Nevertheless, there were 25 deaths during the study period despite rescue procedures. Predictors of life-threatening events were age < 1 year, increased hemodynamic vulnerability, and high procedure complexity. These predictors were then used to calculate standardized life-threatening event ratios, and although most institutions had an expected rate of life-threatening events based on complexity of case mix, one institution showed a significantly greater-than-expected rate of events. These operators reported no greater number of preventable events, suggesting that unmeasured variables may account for the difference. In sum, the present study defines high-risk patients and procedures, the nature of life-threatening events, and the need for appropriate resources to manage these events.

### Risk Modeling

Preprocedure risk stratification can now be significantly enhanced by the development and validation of procedure type risk categories [5] and markers of hemodynamic vulnerability [8]. Taken together with age < 1 year as the third risk predictor, these factors, which form CHARM, can also serve to inform preprocedure risk. In this manner, patients and families may be provided with more specific counseling and informed consent with quantitative estimates of risk. Furthermore, present findings may be used by procedural teams to guide preparation for rescue procedures, such as transcatheter or surgical bail-out, or mechanical support, such as ventricular-assist device or ECMO. Third, these criteria can be used to standardize risk between institutions, operators, or even procedures.

### Quality Improvement

Work from this group has proposed the use of CHARM to provide a method of risk standardization [8] especially with the emphasis on quality improvement in contemporary medicine. The present work provides two important findings in this regard. First, the majority of life-threatening events occurred in the absence of a breach of standard of care, suggesting that operators did not believe that the majority of events were preventable, including the institution with a greater-than-expected rate of life-threatening events. This raises the important question that current understanding of standard of care and adverse event preventability may be insufficiently discriminating or objective to contribute to the present analysis or quality-improvement initiatives. In contrast, overall modification of practice to decrease preventable events may improve overall outcomes. Second, CHARM may provide a means for identifying life-threatening events that require more scrutiny in the quality-improvement process. Specifically, whereas sick patients undergoing complex procedures may be “expected” to have life-threatening events, life-threatening events that occur during cases with lower-risk CHARM characteristics may be “unexpected” and require a more thorough root cause analysis.

Future efforts should be directed toward decreasing institutional as well as global standardized life-threatening event ratio to <1. At least two areas of study may allow progress toward this goal. First, life-threatening events must be divided into those occurring in “expected” circumstances (i.e. sick patient and/or complex procedure) from those occurring in “unexpected” circumstances (i.e. stable patients and/or less complex procedures). Efforts can then be directed toward understanding predictors of emergency rescue procedures and how they can best be prepared before or during a high-risk case. For example, should an ECMO circuit be primed and in the room before or during a case when high-risk features are identified, or should pre-emptive support be initiated? Present results suggest that ready availability and preparation of rescue strategies, including transcatheter bailout, general, and cardiac surgery, and availability of ECMO, are a crucial component to management of life-threatening events and must be considered a prerequisite to performing high-risk procedures in high-risk patients. Similarly, are there patients for whom the risk of life-threatening events is unacceptable? Although the answer to this particular question will always be the collaborative decision of the care team, the operator, and the family of the patient, present findings may help to quantify risk in a manner that better informs this decision.

Second, among patients with unexpected life-threatening events, specific mechanisms of life-threatening events (e.g., transseptal puncture and cardiac perforation, device/coil embolization, etc.) must be studied on a large scale to understand predictors of successful versus adverse outcome and how procedures can be modified and optimized. These future studies will be feasible as more data are acquired through the efforts of this and other projects such as C3PO-Quality Improvement (launching in 2013), the Congenital Cardiovascular Interventional Study Consortium, and the NCDR/IMPACT registry.

### Limitations

Present studies were performed at high- volume centers with a large base of experience both in transcatheter procedures as well as general and cardiovascular surgery. Application of these findings to institutions with heterogeneous backgrounds globally will require a nuanced approach. In addition, due to the low incidence of life-threatening events during myocardial biopsies [4] and hybrid procedures [12], these cases were excluded from this analysis. Finally, although the low mortality rate precluded a meaningful multivariate analysis of predictors of death in this study, our report of the descriptive details herein may assist in hypothesis generation (especially regarding impact of age of the patient) and development of additional studies.

## Conclusion

When compared with recent results from the Cath PCI/NCDR registry where in-hospital mortality rate after percutaneous coronary intervention has been reported as 1.27 %, ranging from 0.65 % in elective PCI to 4.81 % in ST increase myocardial infarction [16], contemporary congenital cardiac catheterization and intervention is safe with a low rate of mortality (0.28 %), and life-threatening events (2.1 %). Although patient and procedural factors have become more complex in the contemporary era, findings define the contribution of age, hemodynamic vulnerability, and procedure type to life-threatening adverse events. By use of CHARM, preprocedural risk can be quantified and allow objective assessment for need of rescue procedures and mechanical support. Likewise, expected outcomes and adverse event rates can be estimated based on this risk model. As such, concern for third-party scrutiny of morbidity and mortality in outcomes should not deter qualified operators from performing high-risk procedures on high-risk patients when clinically indicated in the setting of appropriate preparation of rescue procedures. Findings from this and future studies should be applied to appropriately adjust for procedural risk based on patient and procedural factors.

## Electronic Supplementary Material

Below is the link to the electronic supplementary material.

Supplementary material 1 (DOCX 48 kb)

## References

[1] Andrade JG, Al-Saloos H, Jeewa A, Sandor GGS, Cheung A (2010). Facilitated cardiac recovery in fulminant myocarditis: pediatric use of the Impella LP 5.0 pump. J Heart Lung Transpl.

[2] Bergersen L, Gauvreau K, Jenkins KJ, Lock JE (2008). Adverse event rates in congenital cardiac catheterization: a new understanding of risks. Congenit Heart Dis.

[3] Bergersen L, Gauvreau K, Lock JE, Jenkins KJ (2008). A risk adjusted method for comparing adverse outcomes among practitioners in pediatric and congenital cardiac catheterization. Congenit Heart Dis.

[4] Bergersen L, Marshall A, Gauvreau K (2010). Adverse event rates in congenital cardiac catheterization—multicenter experience. Cathet Cardivasc Interv.

[5] Bergersen L, Gauvreau K, Marshall A (2011). Procedure-type risk categories for pediatric and congenital cardiac catheterization. Catheter Cardiovasc Interv.

[6] Bergersen L, Giroud JM, Jacobs JP (2011). Report from The International Society for Nomenclature of Paediatric and Congenital Heart Disease: cardiovascular catheterisation for congenital and paediatric cardiac disease (part 2—nomenclature of complications associated with interventional cardiology). Cardiol Young.

[7] Bergersen L, Giroud JM, Jacobs JP (2011). Report from The International Society for Nomenclature of Paediatric and Congenital Heart Disease: cardiovascular catheterisation for congenital and paediatric cardiac disease (part 2—nomenclature of complications associated with interventional cardiology). Cardiol Young.

[8] Bergersen L, Gauvreau K, Foerster SR (2011). Catheterization for congenital heart disease adjustment for risk method (CHARM). JACC Cardiovasc Intervent.

[9] Cassidy SC, Schmidt KG, Van Hare GF, Stanger P, Teitel DF (1992). Complications of pediatric cardiac catheterization: a 3-year study. J Am Coll Cardiol.

[10] Committee on Quality of Health Care in America Institute of Medicine (1999). To err is human: building a safer health system.

[11] Hagler DJ (2010). Defining adverse events. Catheter Cardiovasc Interv.

[12] Holzer R, Marshall A, Kreutzer J (2010). Hybrid procedures: adverse events and procedural characteristics—results of a multi-institutional registry. Congenit Heart Dis.

[13] Jenkins KJ, Gauvreau K, Newburger JW, Spray TL, Moller JH, Iezzoni LI (2002). Consensus-based method for risk adjustment for surgery for congenital heart disease. J Thorac Cardiovasc Surg.

[14] Martin GR, Beekman RH, Ing FF (2010). The IMPACT registry improving pediatric and adult congenital treatments. Semin Thorac Cardiovasc Surg Pediatr Card Surg Annu.

[15] Patel MR, Bailey SR, Bonow RO, Chambers CE, Chan PS, Dehmer GJ (2012). ACCF/SCAI/AATS/AHA/ASE/ASNC/HFSA/HRS/SCCM/SCCT/SCMR/STS 2012 appropriate use criteria for diagnostic catheterization: a report of the American College of Cardiology Foundation Appropriate Use Criteria Task Force, Society for Cardiovascular Angiography and Interventions, American Association for Thoracic Surgery, American Heart Association, American Society of Echocardiography, American Society of Nuclear Cardiology, Heart Failure Society of America, Heart Rhythm Society, Society of Critical Care Medicine, Society of Cardiovascular Computed Tomography, Society for Cardiovascular Magnetic Resonance, and Society of Thoracic Surgeons. J Am Coll Cardiol.

[16] Peterson ED, Dai D, DeLong ER (2010). Contemporary mortality risk prediction for percutaneous coronary intervention: results from 588,398 procedures in the National Cardiovascular Data Registry. J Am Coll Cardiol.

[17] Rhodes JF, Asnes JD, Blaufox AD, Sommer RJ (2000). Impact of low body weight on frequency of pediatric cardiac catheterization complications. Am J Cardiol.

[18] Vitiello R, McCrindle BW, Nykanen D, Freedom RM, Benson LN (1998). Complications associated with pediatric cardiac catheterization. J Am Col Cardiol.

[19] Zahn EM, Hellenbrand WE, Lock JE, McElhinney DB (2009). Implantation of the Melody transcatheter pulmonary valve in patients with a dysfunctional right ventricular outflow tract conduit: early results from the U.S. clinical trial. J Am Coll Cardiol.

