# Supplementary material for: Incidence and Management of Life-Threatening Adverse Events During Cardiac Catheterization for Congenital Heart Disease
Source: Pediatr Cardiol. 2013 Jul 31;35(1):140–8. doi: 10.1007/s00246-013-0752-y (PMC3882522; doi:10.1007/s00246-013-0752-y)
Supplement: Supplementary file 1 — Supplementary material 1 (DOCX 48 kb) [file 246_2013_752_MOESM1_ESM.docx]

Supplemental Table 1: Adverse Event Severity

|  | **Severity Level** | **Definition** |
| --- | --- | --- |
| **Low** | **1 – None (very mild)** | No harm, no change in condition, may have required monitoring to assess for potential change in condition with no intervention indicated. |
|  | **2 – Minor** | Transient change in condition, not life threatening, condition returns to baseline, required monitoring, required minor intervention such as holding a medication, or obtaining lab test. |
| **High** | **3 – Moderate** | Transient change in condition may be life threatening if not treated, condition returns to baseline, required monitoring, required intervention such as reversal agent, additional medication, transfer to the intensive care unit for monitoring, or moderate trans-catheter intervention to correct condition. |
|  | **4 – Major** | Change in condition, life threatening if not treated, change in condition may be permanent, may have required an intensive care unit admission or emergent readmit to hospital, may have required invasive monitoring, required interventions such as electrical cardioversion or unanticipated intubation or required major invasive procedures or trans-catheter interventions to correct condition. |
|  | **5 – Catastrophic** | Any death, and emergent surgery or heart lung bypass support (ECMO) to prevent death with failure to wean from bypass support. |

|  | **Definitions of Adverse Event Preventability** |
| --- | --- |
| **Preventable:** | Events where definite breech of standard technique was identified; necessary precautions were not taken; event was preventable by modification of technique or care. |
| **Possibly Preventable:** | Events where definite breech of standard technique was not identified but may have occurred; necessary precautions may not have been taken’ event may have been preventable by modification of technique or care. |
| **Not Preventable:** | Events where no obvious breech of standard technique occurred; necessary precautions were taken; no clearly known alteration in method or care exists to prevent the event. |

# Supplemental Table 2: Definitions of Adverse Event Preventability

**Supplemental Table 3. Definitions of Hemodynamic Vulnerability**

|  | Non Single Ventricle | Single Ventricle |
| --- | --- | --- |
| Mixed Venous Saturation | < 60% | <50% |
| Systemic Ventricle End Diastolic Pressure | ≥ 18mm Hg | ≥ 18mm Hg |
| Systemic Arterial Saturation | <95% | <78% |
| Main Pulmonary Artery Pressure | Systolic ≥ 45mmHg | Mean ≥ 17mm Hg |

**Supplemental Table 4.** Procedure Type Risk Categories

|  | Risk Category 1 | Risk Category 2 | Risk Category 3 | Risk Category 4 |
| --- | --- | --- | --- | --- |
| **Diagnostic Case** | Age ≥ 1 year | Age ≥ 1 month < 1year | Age < 1 month |  |
| **Valvuloplasty** |  | Pulmonary Valve ≥1 month | Aortic valve ≥ 1 month  Pulmonary valve < 1 month  Tricuspid valve | Mitral Valve  Aortic Valve < 1 month |
| **Device or Coil Closure** | Venous collateral  LSVC | PDA  ASD or PFO  Fontan Fenestration  Systemic to Pulmonary Artery collaterals | Systemic Surgical Shunt  Baffle Leak  Coronary Fistula | VSD  Perivalvar leak |
| **Balloon Angioplasty** |  | RVOT  Aorta dilation < 8 ATM | Pulmonary artery < 4 vessels  Pulmonary artery ≥ 4 vessels all < 8 ATM  Aorta > 8 ATM or CB  Systemic Artery (not aorta)  Systemic Surgical Shunt  Systemic to Pulmonary Collaterals  Systemic vein | Pulmonary Artery ≥ 4 vessels  Pulmonary vein |
| **Stent Placement** |  | Systemic vein | RVOT  Aorta  Systemic artery (not aorta) | Ventricular septum  Pulmonary artery  Pulmonary vein  Systemic Surgical Shunt  Systemic pulmonary Collateral |
| **Stent Redilation** |  | RVOT  Atrial Septum  Aorta  Systemic Artery (not Aorta)  Systemic vein | Pulmonary Artery  Pulmonary vein | Ventricular septum |
| **Other** | Myocardial Biopsy | Snare foreign body  Trans-septal puncture | Atrial septostomy  Recanalization of Jailed Vessel in Stent  Recanalization of Occluded Vessel | Atrial Septum Dilation and Stent  Any Catheterization < 4 days after Surgery  Atretic valve perforation |

**Supplemental Table 5.** Description of cases requiring ECMO

| Age | Weight (kg) | Diagnosis | Number of Hemodynamic Indicators | Admission Source | Procedure Type Risk Category | Brief Description of Event | Outcome |
| --- | --- | --- | --- | --- | --- | --- | --- |
| Neonate | 2.5 | Single Ventricle | Hemodynamic vulnerability data not available | Non-Elective | 3 | Following selective left coronary angiography, patient developed complete heart block, and aortic root injection suggested thromboembolism in distal right coronary artery. TPA given in aorta and some improvement in hemodynamics with epinephrine, however, continued deterioration required CPR and subsequent initiation of ECMO. Following hemodynamic stabilization, LV function improved. | Weaned from ECMO |
| Neonate | 1.8 | Single Ventricle | Hemodynamic vulnerability data not available | Non-Elective | 3 | Following case, patient became bradycardic and was placed on ECMO. Three days later ECMO was weaned, however, had recurrent hypotension. As the patient was not a transplant candidate, care was subsequently withdrawn. | ECMO, Death |
| 7 days | 3.5 | Two Ventricle | ≥2 | Emergent | No Risk Designation Assigned | Patient with profound LV dysfunction with severe hypotension, cyanosis, bradycardia transferred to cath lab from ER. Due to ongoing hemodynamic instability, ECMO successfully initiated. | Weaned from ECMO |
| 7 days | 3.7 | Single Ventricle | ≥2 | Non-Elective | 4 | Patient status post RV-PA conduit with plan for RPA stent. With 5F sheath across tricuspid and RV-PA conduit, became hypotensive and bradycardic requiring pacing and epinephrine, even after sheath removed and stent deployed. CPR initiated with good effect until ECMO successfully initiated. Following stabilization on ECMO, addition angiography demonstrated patency of PAs and coronary arteries. | Weaned from ECMO |
| 7 days | 3.7 | Two Ventricle | Hemodynamic vulnerability data not available | Non-Elective | 4 | Critically ill infant in low cardiac output syndrome on maximal inotropic therapy brought to cath lab, became bradycardic and hypotensive requiring chest compressions. Endotracheal tube had migrated to right mainstem bronchus. After additional epinephrine and calcium, regained perfusing rhythm, tube adjusted, and patient placed on ECMO. Atrial septal dilation performed following stabilization. | Weaned from ECMO |
| 7 days | 4.1 | Two Ventricle | Hemodynamic vulnerability data not available | Non-Elective | 3 | Following lobar PA and MAPCA stenting, aortic angiogram revealed ostial left main coronary stenosis, and RCA angiogram performed. Due to deep cannulation of the RCA, wide QRS complexes and ST elevations were seen with hypotension, and pressors were initiated. The patient was successfully initiated on ECMO. Following stabilization, aortic angiogram demonstrated RCA spasm, and as a result, the coronary was wired and angioplasty performed with improvement. The left coronary could not be cannulated and therefore a atrial septostomy and balloon dilation of coarctation were performed for palliation. | Weaned from ECMO |
| 25 days | 2.9 | Single Ventricle | ≥2 | Emergent | 4 | Single ventricle patient with stenotic BT shunt cyanotic on arrival arrested during initial hemodynamic cath, successfully placed on ECMO. Once stabilized, BT shunt successfully stented. Nevertheless, subsequently expired. | ECMO, Death |
| 2 months, 16 days | 4 | Two Ventricle | ≥2 | Non-Elective | 3 | Following angioplasty of right pulmonary collateral, patient became bradycardic due to severe spasm of the collateral. Nitric oxide, atropine, and epinephrine initiated with good effect. Recurrent spasm occurred near the end of the case and patient became bradycardic and hypotensive refractory to pressors, and patient cannulated on ECMO during on ongoing CPR. | Weaned from ECMO |

**Supplemental Table 5 (continued).** Description of cases requiring ECMO

| Age | Weight (kg) | Diagnosis | Number of Hemodynamic Indicators | Admission Source | Procedure Type Risk Category | Brief Description of Event | Outcome |
| --- | --- | --- | --- | --- | --- | --- | --- |
| 4 months | 4.9 | Single Ventricle | ≥2 | Non-Elective | 3 | During case, patient required increasing inotropic support and transfusion, however, flash pulmonary edema with blood from ETT tube when catheters entered LV through both aortic and mitral valves. CPR initiated and patient cannulated to ECMO. | Weaned from ECMO |
| 10 months, 10 days | 7.7 | Two Ventricle | 1 | Elective | 4 | During a series of pulmonary angioplasties, the wire was repositioned across the RVOT, and patient developed hypotension and bradycardia a poor end-tidal CO2 which persisted despite epinephrine and removal of all equipment from the heart. CPR given for 5 minutes with return of perfusion, however, 10 minutes later became severely bradycardic and hypotensive without response to CPR. Placed on ECMO after 60 minutes of CPR, however, noted to have severe anoxic brain injury. ECMO discontinued the day after and patient died. | ECMO, Death |
| 1 year, 1 month | 9.5 | Two Ventricle | ≥2 | Non-Elective | 1 | Patient initially did well after stent placement in the left main coronary artery, however, four days later, acutely decompensated with bradycardia and hypotension with subsequent degeneration to ventricular fibrillation while preparing in the cath lab for cannulation on ECMO. After stabilization on ECMO, coronary angiography demonstrated a stent thrombosis, but flow to LAD and circumflex. As a result, the patient was taken to the OR for repair, but subsequent course was complicated by bleeding and stroke. The family decided to withdraw, and patient expired. | ECMO, Death |
| 5 years | 16 | Two Ventricle | 1 | Non-Elective | No Risk Designation Assigned | During balloon dilation of the left coronary artery, patient became bradycardic and hypotensive, intermittently requiring CPR and epinephrine with good effect, however, ultimately required initiation of ECMO during a period of apparent cardiac standstill. This resolved by the time procedure completed and patient transferred from cath lab. | Weaned from ECMO |
| 7 years | 17 | Two Ventricle | Hemodynamic vulnerability data not available | Elective | 3 | During left PA angioplasty, air noted in RV and blood in mouth and nose of patient. Balloon reinflated at suspected tear site and patient emergently intubated. However, patient then went into complete heart block and hypotension. CPR initiated and transvenous pacing catheter placed successfully. Nevertheless, transfemoral ECMO cannulation initiated. Following stabilization, the patient was taken to the operating room for LPA repair. | ECMO, Transferred to OR, Weaned from ECMO |
| 12 years | 34 | No Structural Disease | ≥2 | Elective | 1 | Patient with restrictive cardiomyopathy underwent diagnostic catheterization with endomyocardial biopsy, without complications. After sheathes removed, anesthesia reversed, patient went into complete heart block with unsuccessful transvenous pacing, and ECMO cannulation attempted during CPR. Inadequate flows obtained despite attempt to cannulate from femoral venous access as well. Resuscitation discontinued after three hours and patient expired. | ECMO, Death |
| 14 years | 55 | No structural heart disease | Hemodynamic vulnerability data not available | Non-Elective | 1 | Patient with ventricular arrhythmias brought to cath lab for planned pacing and endomyocardial biopsy, however, degenerated to ventricular fibrillation unresponsive to lidocaine or pacing. ECMO initiated successfully for continued incessant ventricular tachycardia, and right heart cath and biopsy performed following stabilization. Patient found to have severe myocarditis. Transferred, but subsequently expired. | ECMO, Transferred, Death |
| 38 years | 57 | Two Ventricle | ≥2 | Elective | 3 | During the course of pulmonary artery stenting and Melody valve implantation, selective coronary angiography was complicated by coronary artery dissection resulting in cardiac arrest requiring CPR and initiation of ECMO. Coronary was subsequently recanalized and patient weaned off ECMO, but patient expired later during hospitalization. | ECMO, Death |

**Supplemental Table 6.** Description of cases with Catastrophic Adverse Events (Category 5)*

| Age | Weight (kg) | Diagnosis | Number of Hemodynamic Indicators | Admission Source | Procedure Type Risk Category | Brief Description of Event | Outcome |
| --- | --- | --- | --- | --- | --- | --- | --- |
| Neonate | 1.8 | Single Ventricle | Hemodynamic vulnerability data not available | Non-Elective | 3 | Following case, patient became bradycardic and was placed on ECMO. Three days later ECMO was weaned, however, had recurrent hypotension. As the patient was not a transplant candidate, care was subsequently withdrawn. | ECMO, Death |
| Neonate | 1.4 | Single Ventricle | ≥2 | Emergent | No Risk Designation Assigned | Outside hospital neonate HLHS intact atrial septum (ex- 30 week gestation) transferred directly to cath lab. Transhepatic access obtained, however, as sheath introduced into RA, case complicated by complete heart block. Despite return of spontaneous rhythm, unable to enter LA (<5mm). Patient transferred to unit for comfort care and expired 30 minutes later. | Death |
| Neonate | 3.3 | Two ventricle | Hemodynamic vulnerability data not available | Emergent | 3 | Outside hospital transfer, dTGA intact atrial septum, profound hypoxemia, pH6.6. Atrial septal puncture successful, but complicated by perforation, SVT, and VF. Second puncture successful and improvement in saturations after static balloon dilation. On transfer to bed, became hypotensive, large pericardial effusion noted on echo and successfully drained, however, following transfer to ICU, patient expired an hour later due to persistent hemorrhage, coagulopathy, and acidosis. | Death |
| Neonate | 2 | Two ventricle | 1 | Emergent | 3 | Following transfer from outside hospital, prior to procedure, patient became hypotensive/bradycardic and CPR initiated. Chest tube was placed for concern of tension pneumothorax, and a pacing catheter was placed in the right atrial appendage. Warming lights and blankets applied due to hypothermia (31.2C). Nevertheless, patient remained hypotensive despite resumption of sinus rhythm. Support withdrawn after family discussion. | Death |
| Neonate | 1.7 | Single Ventricle | ≥2 | Emergent | No Risk Designation Assigned | Attempted radiofrequency septal perforation resulted in entering pericardial space twice, and on third attempt, air was aspirated from catheter. Patient then became hypotensive and bradycardic. Chest tubes placed bilaterally with 20cc of blood removed from left, but continued cardiac arrest without response to resuscitation and efforts discontinued. | Death |
| Neonate | 3.5 | Single Ventricle | Hemodynamic vulnerability data not available | Emergent | 4 | Following delivery by Caesarean section in adjoining cath lab, HLHS with restrictive atrial septum brought to table with hypoxia, hypotension, and acidosis. Hepatic access obtained after femoral access unsuccessful, and needle septostomy resulted in perforation, but treated by successful pericardiocentesis. Second attempt successful, but resulted in heart block and hypotension, requiring CPR during stent placement. Patient was unable to be resuscitated and expired in the lab. | Death |
| Neonate | 3.1 | Single Ventricle | Hemodynamic vulnerability data not available | Non-Elective | 3 | Hypotension and tachycardia followed by bradycardia after initial resuscitative efforts with degeneration to electromechanical dissociation. Pericardial effusion noted with successful drainage, however, long resuscitation with intentional hypoventilation to improve forward cardiac output. Following discussion with family, decision made to move to comfort care and patient expired in ICU. | Death |

**Cases from Supplemental Table 5 who subsequently expired despite ECMO support are also listed in this table.*

**Supplemental Table 6 (continued).** Description of cases with Catastrophic Adverse Events (Category 5)

| Age | Weight (kg) | Diagnosis | Number of Hemodynamic Indicators | Admission Source | Procedure Type Risk Category | Brief Description of Event | Outcome |
| --- | --- | --- | --- | --- | --- | --- | --- |
| Neonate | 1.5 | Single Ventricle | Hemodynamic vulnerability data not available | Emergent | 2 | Chiba needle used to puncture atrial septum and wired into RUPV with successful static balloon dilation followed by a second balloon inflation. Patient became bradycardic at this point and successful pericardiocentesis performed with removal of 5cc of dark blood, however chest compressions initiated after persistent bradycardia, and left atrial perforation noted. Reattempt at septal perforation made, however, the wire advanced into pericardial space again, and patient subsequently expired despite attempts at resuscitation. | Death |
| Neonate | 3 | Two Ventricle | 1 | Emergent | 3 | Neonate with dTGA transported from outside nursery markedly hypoxemic and acidotic with no response to PGE. Initial pH6.8, but balloon septostomy performed 16 minutes after arrival in lab. However, patient did not recover despite full resuscitation over an hour, and expired in the lab. | Death |
| 10 days | 1.6 | Two Ventricle | ≥2 | Non-Elective | 3 | Following the end of the procedure, the patient became hypotensive and tachycardic. Melanic stools were found under the drapes. The patient was given blood and dopamine, and empiric adjustment of the endotracheal tube was performed with minimal improvement. Hypotension continued to progress, and epinephrine boluses given with some stabilization, but on arrival to the ICU, she continued to have progressive hypotension without response to pressors or volume. Bedside ultrasound demonstrated no pericardial or pleural effusion, but the patient subsequently expired following no response to resuscitation. | Death |
| 25 days | 2.9 | Single Ventricle | ≥2 | Emergent | 4 | Single ventricle patient with stenotic BT shunt cyanotic on arrival arrested during initial hemodynamic cath, successfully placed on ECMO. Once stabilized, BT shunt successfully stented. Nevertheless, subsequently expired. | ECMO, Death |
| 2 months, 27 days | 4.3 | Single Ventricle with Shunt | ≥2 | Not Elective | No Risk Designation Assigned | PDA instent restenosis and possible thrombus with severe progressive hypoxia, metabolic and respiratory acidosis and recent upper respiratory illness. Unable to cross stent, and due to progressive clinical decline, transferred emergently to OR for modified Blalock Taussig shunt. Subsequently expired. | Death |
| 4 months, 18 days | 5.5 | Single Ventricle | Hemodynamic vulnerability data not available | Non-Elective | 2 | Critically ill patient already in pulseless arrest on arrival to the cath lab. Single angiogram performed through central line demonstrated a patent bidirectional Glenn and a moderate-large veno-venous collateral. Otherwise resuscitation efforts unsuccessful and the patient expired later in the ICU. | Death |
| 5 months, 3 days | 5.9 | Two Ventricle | 1 | Elective | 2 | Patient with history of critical pulmonic stenosis who had undergone valvuloplasty, now with dilated LV with plan for PDA coil. Following partial deployment of coil, had suboptimal appearance and the coil was brought back with the catheter with a plan for removal from the femoral venous sheath. However, the coil subsequently embolized to RA, across PFO and down aorta into the SMA. Transcatheter removal unsuccessful, so the patient was taken to the OR where vascular surgery removed the coil, however intestines appeared dusky. On reexploration at 48 hours, the entire small bowel appeared infarcted, and the patient was given comfort care and subsequently expired. | Death |

**Supplemental Table 6 (continued).** Description of cases with Catastrophic Adverse Events (Category 5)

| Age | Weight (kg) | Diagnosis | Number of Hemodynamic Indicators | Admission Source | Procedure Type Risk Category | Brief Description of Event | Outcome |
| --- | --- | --- | --- | --- | --- | --- | --- |
| 6 months, 17 days | 5.3 | Two Ventricle | Hemodynamic vulnerability data not available | Elective | 3 | Following balloon aortic valvuloplasty, patient was noted to have decreased femoral pulse following the procedure. Had fever and irritability with subsequent aspiration of formula and cardiac arrest on the ward. Resuscitation unsuccessful and the patient expired. On autopsy, a large retroperitoneal hemorrhage was found on the access side. | Death |
| 10 months, 10 days | 7.7 | Two Ventricle | 1 | Elective | 4 | During a series of pulmonary angioplasties, the wire was repositioned across the RVOT, and patient developed hypotension and bradycardia a poor end-tidal CO2 which persisted despite epinephrine and removal of all equipment from the heart. CPR given for 5 minutes with return of perfusion, however, 10 minutes later became severely bradycardic and hypotensive without response to CPR. Placed on ECMO after 60 minutes of CPR, however, noted to have severe anoxic brain injury. ECMO discontinued the day after and patient died. | ECMO, Death |
| 11 months, 2 days | 5.5 | Isolated Defect | ≥2 | Emergent | 4 | Moribund patient status post lung transplant with prior pulmonary vein stenosis requiring balloon dilation, presented with acute respiratory distress and hemodynamic compromise, requiring epinephrine and norepinephrine en route to cath lab. Dilation of ASD performed successfully, but during ballon dilation of LLPV, bubbles see in LA. Gradual decline in heart rate and blood pressure ultimately requiring CPR for eight minutes with recovery, but recurrent decline once transferred back to ICU. No further intervention performed and patient expired. | Death |
| 1 year, 1 month | 9.5 | Two Ventricle | ≥2 | Non-Elective | 1 | Patient initially did well after stent placement in the left main coronary artery, however, four days later, acutely decompensated with bradycardia and hypotension with subsequent degeneration to ventricular fibrillation while preparing in the cath lab for cannulation on ECMO. After stabilization on ECMO, coronary angiography demonstrated a stent thrombosis, but flow to LAD and circumflex. As a result, the patient was taken to the OR for repair, but subsequent course was complicated by bleeding and stroke. As a result, the family decided to withdraw, and patient expired. | ECMO, Death |
| 1 year, 5 months | 5 | Two Ventricle | ≥2 | Non-Elective | 4 | Prior to arrival in cath lab, patient arrested twice in ICU, on first postoperative day from cardiac surgery. Underwent catheterization without complications, but after procedure had bradycardic arrest and could not be resuscitated. | Death |
| 12 years | 34 | No Structural Disease | ≥2 | Elective | 1 | Patient with restrictive cardiomyopathy underwent diagnostic catheterization with endomyocardial biopsy, without complications. After sheathes removed, anesthesia reversed, patient went into complete heart block with unsuccessful transvenous pacing, and ECMO cannulation attempted during CPR. Inadequate flows obtained despite attempt to cannulate from femoral venous access as well. Resuscitation discontinued after three hours and patient expired. | ECMO, Death |
| 14 years | 55 | No structural heart disease | Hemodynamic vulnerability data not available | Non-Elective | 1 | Patient with ventricular arrhythmias brought to cath lab for planned pacing and endomyocardial biopsy, however, degenerated to ventricular fibrillation unresponsive to lidocaine or pacing. ECMO initiated successfully for continued incessant ventricular tachycardia, and right heart cath and biopsy performed following stabilization. Patient found to have severe myocarditis. | ECMO, Death |

**Supplemental Table 6 (continued).** Description of cases with Catastrophic Adverse Events (Category 5)

| Age | Weight (kg) | Diagnosis | Number of Hemodynamic Indicators | Admission Source | Procedure Type Risk Category | Brief Description of Event | Outcome | |  |
| --- | --- | --- | --- | --- | --- | --- | --- | --- | --- |
| 15 years | 63 | Pulmonary Hypertension | ≥2 | Non-Elective | 4 | Patient with pulmonary hypertension underwent successful RF atrial septal puncture and stenting. When moved to bed for transport, saturations dropped to 60s and became hypotensive, requiring epinephrine infusion. CPR then initiated given progressive hypotension, however, patient could not be resuscitated. | | Death | |
| 30 years | 63 | Single Ventricle | ≥2 | Emergent | No Risk Designation Assigned | Severely ill patient became hypotensive (systolic 50s) and bradycardic during initial access despite epinephrine. CPR initiated, and empiric pericardiocentesis performed without improvement. Pacing attempted, without success, and degenerated to VF requiring DC cardioversion. However resuscitation remained unsuccessful and the patient expired. | | Death | |
| 38 years | 57 | Two Ventricle | ≥2 | Elective | 3 | During the course of pulmonary artery stenting and Melody valve implantation, selective coronary angiography was complicated by coronary artery dissection resulting in cardiac arrest requiring CPR and initiation of ECMO. Subsequently expired. | | ECMO,  Death | |
| 39 years | 80 | Pulmonary Hypertension | 1 | Non-Elective | 3 | Atrial septal puncture with needle found to be in anterior pericardial reflection. Sheath left in place, and second access used to place septostomy. Heparin reversed, and pericardiocentesis performed with 390 cc of serosanguinous drainage. Once perforating sheath removed, pericardial effusion reaccumulated, and resuscitation required multiple pressors and patient taken to OR for repair. No atrial perforation identified, however, perforation in RV anterior wall noted and repaired. Given continued hemodynamic decline, evidence of significant CNS injury, patient was made DNR and subsequently expired. | | Death | |
